# Polyurethane reinforced with micro/nano waste slag as a shielding panel for photons (experimental and theoretical study)

**DOI:** 10.1038/s41598-024-60482-z

**Published:** 2024-05-08

**Authors:** Ahmed M. El-Khatib, Mahmoud I. Abbas, Mohamed E. Mahmoud, Mohammed Fayez-Hassan, Mamdouh H. Khalil, Ahmed Abd El Aal

**Affiliations:** 1 Physics Department, Faculty of Science, Alexandria University, Alexandria, 21511 Egypt; 2 Chemistry Department, Faculty of Science, Alexandria University, P.O. Box 426, Ibrahimia, Alexandria, 21321 Egypt; 3 Experimental Nuclear Physics, Nuclear Research Center, Egyptian Atomic Energy Authority, Inshas, Cairo, 13759 Egypt; 4 Alex Steel, Desert Road Km 21 Merghm, Alexandria, 23722 Egypt; 5 Alex Form, Desert Road Km 21 Merghm, Alexandria, 23722 Egypt

**Keywords:** Polyurethane, Nano composite, Waste iron slag, Shielding parameters, Thermogravimetric analysis, X-ray diffraction, Fourier transform infrared analysis, Nuclear protection, Monte Carlo, FLUKA, Chemistry, Materials science, Nanoscience and technology

## Abstract

This study not only provides an innovative technique for producing rigid polyurethane foam (RPUF) composites, but it also offers a way to reuse metallurgical solid waste. Rigid polyurethane (RPUF) composite samples have been prepared with different proportions of iron slag as additives, with a range of 0–25% mass by weight. The process of grinding iron slag microparticles into iron slag nanoparticles powder was accomplished with the use of a high-energy ball mill. The synthesized samples have been characterized using Fourier Transform Infrared Spectroscopy, and Scanning Electron Microscope. Then, their radiation shielding properties were measured by using A hyper-pure germanium detector using point sources 241Am, 133 BA, 152 EU, 137Cs, and 60Co, with an energy range of 0.059–1.408 MeV. Then using Fluka simulation code to validate the results in the energy range of photon energies of 0.0001–100 MeV. The linear attenuation coefficient, mass attenuation coefficient, mean free path, half-value layer and tenth-value layer, were calculated to determine the radiation shielding characteristics of the composite samples. The calculated values are in good agreement with the calculated values. The results of this study showed that the gamma-ray and neutron attenuation parameters of the studied polyurethane composite samples have improved. Moreover, the effect of iron slag not only increases the gamma-ray attenuation shielding properties but also enhances compressive strength and the thermal stability. Which encourages us to use polyurethane iron-slag composite foam in sandwich panel manufacturing as walls to provide protection from radiation and also heat insulation.

## Introduction

Rigid polyurethane foam (RPUF) is a type of polymer insulation material with low thermal conductivity, low density, high specific strength, and easy fabrication that is extensively utilized in construction, automobiles, and home appliances. Because of its excellent thermal insulation properties, rigid polyurethane foam (RPUF) is commonly utilized in exterior wall insulation materials.

Researchers have been interested in polyurethane (PU) and PU-based composites due to their promising properties. By adding different kinds of fillers to the PU foam, their functional properties can be controlled. Various nanofillers, such as graphene is used to improve the performance of thermal insulation foams^1^, nano clay is used to improve radiation protection against photons^2^, nano- and micro-silica fillers are used to improve compressive strength and density^3^, nano-SiO_2_ is used to improve their structure and mechanical properties^4^, and Pb is used to improve gamma-ray nuclear shielding^5^and etc.

Every year, about 100–130 million tons of iron slag (IS) are produced as byproducts of steel production in Basic Oxygen Furnace (BOF). The average quantity of iron slag is about 80–120 kg per ton of steel^6–8^. As a result, they use of iron slag (IS) in the concrete industry is gaining popularity^9^. Iron slag (IS) is rich in metal oxides such as CaO, SiO_2_, and Al_2_O_3_. These compounds exhibit excellent catalytic carbonization and smoke suppression effects in combustion and thus have potential applications in fire-retarding fields^10,11^.

The protection of workers and public against the hazards of radiation by using low-cost materials and especially waste materials is a crucial target for researchers^12–17^.

As a result, finding new prospective IS fields of application and using IS with high added value is critical in the metallurgical industry. The aim of this study was to use two main materials, which are polyurethane chemicals and metal waste from Basic Oxygen Furnace (BOF) as iron slag. The target of the new compounds was to offer a fresh approach to using (IS) as a flame retardant in fire safety engineering. Our results showed that adding (IS) might enhance the (RPUF) composites' high temperature thermal stability. Another objective of the experiments was to examine the efficiency of ISMPs and ISNPs on gamma-ray attenuation was to examine the efficiency of ISMPs and ISNPs on gamma-ray attenuation.

Based on the experimental results the LAC was determined for all analyzed composite samples, and by knowing the samples densities, we Calculating the MAC, to confirm the experimental results we use online software XCOM software and FLUKA software were used to establish a satisfactory agreement can be achieved between the experimental and XCOM results. We perform the Mont Carlo Fluka simulation because FLUKA is a fully integrated particle physics Monte Carlo simulation package. It has many applications in experimental physics and engineering, Shielding Aspects of Accelerators, Targets, and Irradiation Facilities, detectors, dosimetry, medical physics and radio-biology. Moreover, Fluka is offline CERN software. It can use the modern neutron and photon libraries BROND-3.1, ENDF-VIII0, JEFF-3.3, and JENDEL- 4.0. As we can see, it can provide and can give accurate results about the collective attenuation of photons. The Monte Carlo simulation in particular distinguishes itself from other programs by showing high-quality results at very small photon energy. It is better than other software because it gives results over a wide range of photon energy with a very narrow energy interval and is very useful in predicting accurate iterated results for any photon energy interval which passes through a sequence of instructions simulations being repeated until a specific end result is achieved. In Fig. 17. The energy range 0.0001–0.1 MeV the simulation gave very detailed results, and many energies were clearly monitored, and this is unique in using the FLUKA program.

In general, a primary radiation (in this case 100 MeV proton beam) for producing radioisotopes makes several secondary radiations such as, photons neutrons, protons, and so on. Each radiation has to be shielded from workers who are operating and maintaining the machine with a proper shielding method. Therefore, a shielding calculation for the designed configuration of target room has to be carried out before the facility construction with the aim of good shielding and saving the expenses.

The theoretical calculations of the shielding parameters using Monte Carlo software’s will contribute to enhance the characteristics of the proposed shielding materials. Fluka software is one of these software’s used to design and calculate the shielding parameters of any composite material^18–24^.

## Materials and methods

### Materials

The chemical compounds needed to create rigid polyurethane foam were acquired from BASF chemical company, Schwarzheide, Germany. Elastopir 1132/509/0 is the primary raw material for polyol, polymeric Diphenylmethane Di isocyanate (IsoPMDI 92410), Reactive blowing Catalyst KX 133 catalyst, Trimerization Catalyst KX 340/1, n-Pentane ,Toluene rectified were supplied from Piochem chemical company, Cairo, Egypt. The micro size iron slag used in this study was collected from the dumping yard of Ezz Dekheila Steel Co, Alexandria, Egypt.

### Preparation of nano IS using a high energy ball mill (HEBM) machine

Powder was produced by high-energy planetary ball milling (Photon—model PH-BML 912). Planetary ball milling the particles of iron slag from micro size to nanosized, a planetary ball performs the grinding process in a zirconium chamber, and zirconium balls of 19 mm and 10 mm diameter. The total duration of milling is 10 h, with an interval of 30 min every two hours. The rotation speed of the planet carrier is 600 rpm. The ball mill is loaded with a ball-to-powder weight ratio of 1:1. The chemical toluene liquid is used as the medium to reduce heat generation and avoid agglomeration. The milled powder is taken out and kept in the dryer at 60 °C to remove the toluene liquid content^5^.

### Composite preparation (metal-polymer mixing)

**Figure 1:**
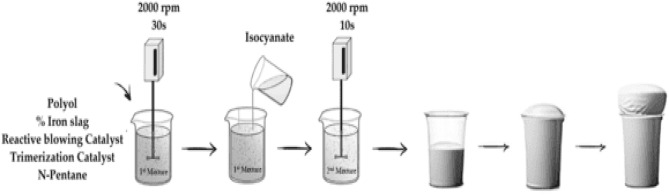
Polymer preparation scheme.

Rigid polyurethane foam composite prepared with different ratios of iron slag filler (0 wt%, 5 wt%, 15 wt%, and 25 wt%) from the total chemical amount. The mixture was prepared in a cup using free-rising methods, as shown in Fig. 1 and according to the quantities in Table 1.

**Table 1:** The compositions of RPUFs samples (all quantities in gm).

Component	PUF	ISMPs	ISMPs	ISMPs	ISNPs	ISNPs	ISNPs
		5%	15%	25%	5%	15%	25%
ISMPs	0.00	4.30	13.00	21.70	0.00	0.00	0.00
ISNPs	0.00	0.00	0.00	0.00	4.30	13.00	21.70
Polyol	25.00	23.80	21.30	18.80	23.80	21.30	18.80
Isocyanate	57.50	54.60	48.90	43.10	54.60	48.90	43.10
Catalyst	0.73	0.70	0.60	0.50	0.70	0.60	0.50
Additive	0.60	0.60	0.50	0.50	0.60	0.50	0.50
Pentane	3.13	3.00	2.70	2.30	3.00	2.70	2.30

The first step to producing RPUF composite is to mix IS powder with the polyol by mechanical stirrer for 30 s, then mix the mixture with a reactive blowing catalyst, a trimerization catalyst, and a physical blowing agent. Finally, isocyanate is added and stirred for 10 s, the test is done at room temp. After curing for 24 h, the samples will be ready for testing^5^.

### Characterization techniques

#### Energy-dispersive X-ray spectroscopy (EDX)

The elements present and their ratio in the slag powder were measured using (JSM-IT200 Series), Nieuw—Vennep, Netherlands.

#### Transmission electron microscope (TEM)

The size of the aggregates was analyzed by employing the TEM (JEM2100F, JEOL, Tokyo, Japan) at 200 kV. The sample was prepared by dispersing the powder in ethanol by ultrasonic vibration on a Cu grid.

#### Scanning electron microscopy (SEM)

The morphology of the composite samples was observed with a scanning electron microscope (Hitachi TM3030, Japan). The magnification was 40 times with a 15 kV accelerating voltage. The images of surface topography were analyzed in order to obtain the cell diameter.

### Apparent density measurement

The density of the samples was measured as per EN 1602^25^. The samples were cut into cylindrical shapes with a height of 80 ± 1 mm and a radius of 40 ± 1 mm. The dimensions of each sample were measured using a digital Vernier caliper. A Precisa Ls 1220 M electronic balance from Dietikon, Switzerland, was used to measure the masses of the investigated samples.

### Compressive strengthen test

ASTM D1621-1^26^ was used to measure compressive Strengthen under compression using a DEVOTRANS DVT FU/DLC in Istanbul, Turkey. RPUF composite samples were compressed from 10 to 50% of the total thickness, at a rate of 3 m/min.

#### Thermo-gravimetric analysis (TGA)

The thermogravimetric analyzer (TG 209F3) from NETZSCH in Selb, Germany, was used to investigate the thermal stability behavior of the composite samples. For the combustion experiment, roughly 10 mg of material was placed into a platinum crucible and heated at a rate of 20 K/min at temperatures ranging from 40 to 700 °C. The furnace environment was made up of synthetic air (VN2 = 80%, VO2 = 20%) and flowed at a rate of 40 ml/min.

#### Diffraction analysis (XRD) analysis

The chemical composition and structure of the samples were characterized using advanced X-ray diffraction analysis on the D2 Phaser 2nd Gen Bruker Model, Billerica, MA, USA. XRD experiments were conducted on every composite sample. All analyses were conducted at a temperature of room temperature. The test was performed with Cu-Ka XRD radiation, at 30 kV and 30 mA, acceleration currency 10 mA, and scan range 10–70 degrees.

#### Fourier-transform infrared spectroscopy (FTIR) analysis

Fourier’s Infrared Transform Spectroscopy (FTIR) is a well-developed analytical method for hydrogen bonding analysis and phase separation phenomena^27^. A Shimadzu dxp 400 (Kyoto, Japan) was used to record the spectra of iron slag. The analytical range is about 400–4000 cm^−1^.

### Gamma‑ray acquisition setup

**Figure 2:**
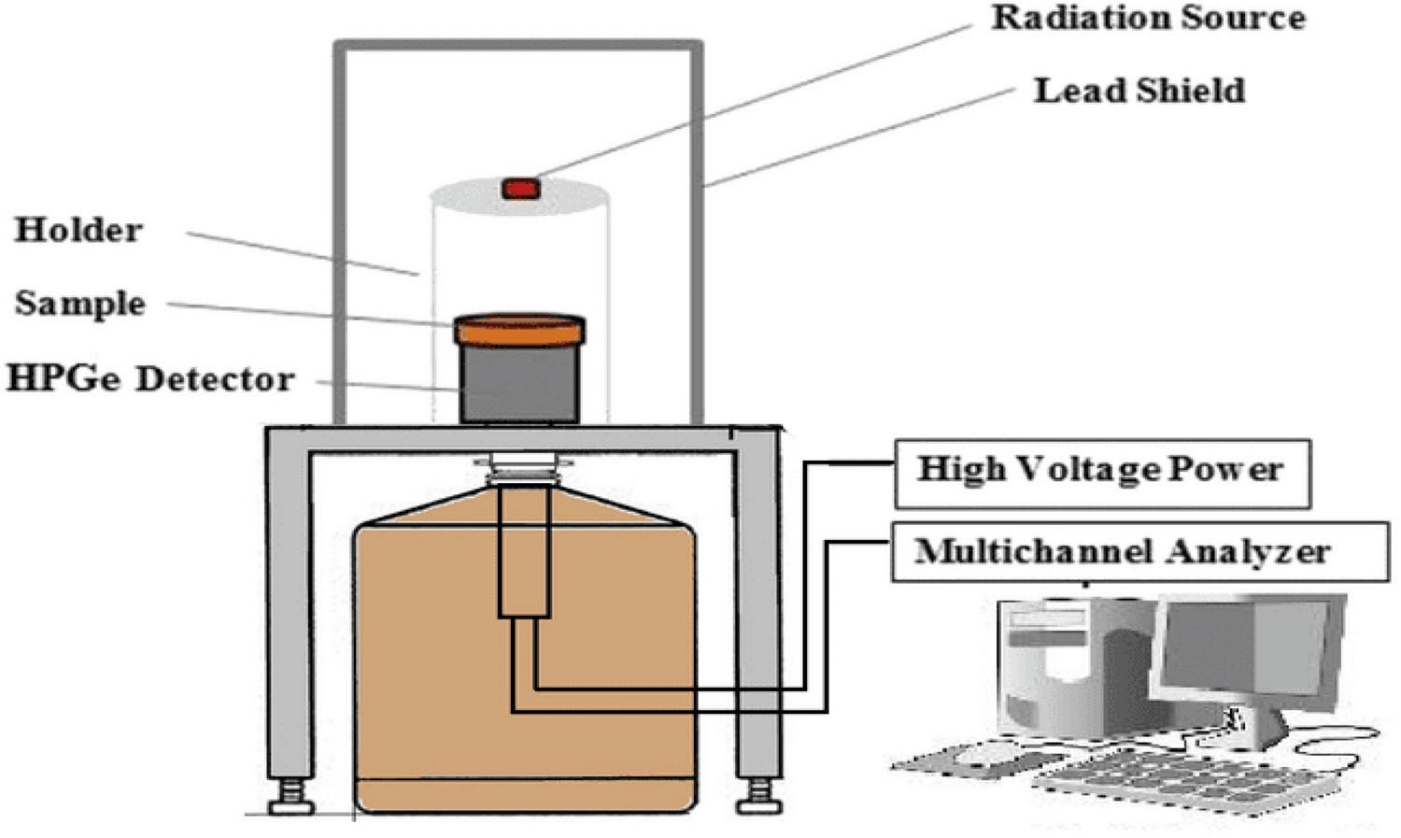
Experimental setup for a (HPGe) detector.

The experimental study is being conducted at Alexandria University, Faculty of Science, Physics Department, Prof. Dr. Younis. S. Selim Laboratory for Radiation Physics. In the present work, shielding experimental results of the analyzed samples were tested by using a calibrated P-type hyper-pure germanium cylindrical detector (HPGe) from Canberra, Atlanta, Georgia, United States, standard, and the gamma ray radiation point sources were used (241Am, 133Ba, 152Eu, 137Cs, and 60Co), which were obtained from the Physikalisch-Technische Bundesanstalt (PTB) in Braunschweig, Berlin, Germany" point sources gamma-ray emitters according to Table 2. The photon energy was selected between 0.059 and 1.408 MeV^28^. The photons that emerged from the sample interacted with the detector crystal^29^. The radioactive sources were placed at 508.67 mm from the detector surface to get a narrow beam of photons, to minimize the effect of detector dead time to be below 1% and also to ignore the summing effects^24,30^. The experimental setup shown in Fig. 2. The detector was calibrated to get the best geometry for the measurements, and the free sample intensity of the present sources was measured (I_o_). Following this, the measurements were done for nano and micro foam composite samples to calculate the intensity (I) at a specific thickness (x). From these values, the experimental LAC can be calculated. The spectra acquired by the gamma analyzer software The program automatically searched for peaks and calculated their areas. We also adjusted the peak fit interactively when needed to minimize the residuals and error. We used the program to analyze the data. To validate the validity of these experimental data, the MAC was calculated using the sample densities and the LAC for each composite sample. To validate the experimental results, we used XCOM software and FLUKA programs, which showed a satisfactory agreement between the experimental, XCOM results and FLUKA results.

**Table 2:** Photon energies, and half-life time for all radioactive sources for the all radionuclides used in this work.

PTB-nuclide	Energy (MeV)	Current Activity (KBq)	Emission probability %	Half-life (days)
241Am	59.52 × 10^−3^	252.77	35.90	157,861.05
133Ba	80.99 × 10^−3^	101.24	34.10	3847.91
152Eu	121.78 × 10^−3^	133.38	28.40	4943.29
	244.69 × 10^−3^		7.490	
	344.28 × 10^−3^		26.00	
	778.9 × 10^−3^		12.96	
	964.13 × 10^−3^		14.00	
	1408.01 × 10^−3^		20.87	
137Cs	661.66 × 10^−3^	271.40	85.21	11,004.98
60Co	1173.23 × 10^−3^	28.86	99.90	1925.31
	1332.5 × 10^−3^		99.98

### Theoretical background

#### Linear attenuation coefficient (LAC)

The LAC (μ) describes the probability of gamma ray interaction (absorption or scattering) per unit thickness of the material. calculated by using Beer-Lambert’s law Eq. (1)^31^

1 $${\text{LAC}} = ~\upmu = \frac{1}{{\text{t}}}\ln \left( {\frac{{{\text{I}}_{{\text{0}}} }}{{\text{I}}}} \right)$$

where: I_0_ is the photon initial intensity. I is the photon-transmitted intensity. t material thickness (cm) μ is the LAC (cm^-1^).

#### Mass attenuation coefficient (MAC)

MAC it represents the probability of interaction per unit mass of the material (cm^2^/g) calculated using Eq. (2)^32^

2 $${\text{MCA }} = \upmu/ \uprho$$

where:

- μ is the LAC (cm^-1^)
- ρ is the material density (g/cm^3^)

Using the XCOM program^33–35^, The theoretical MAC was analyzed and contrasted with experimental values.

#### Mean Free Path (MFP)—λ

MFP represents the average distance a gamma ray travels before interacting with the material. It's the reciprocal of the LAC (cm), calculated using Eq. (3).

3 $${\text{MFP = }}\frac{{1}}{{\upmu }}$$

#### Half-value layer (HVL) and tenth-value layer TVL

HVL and TVL is the thickness of a material required to reduce the initial gamma ray intensity by half and tenth respectively.

The mathematical expression of TVL and HVL are given by the following Eqs. (4–5).

4 $${\text{TVL = }}\frac{{\text{Ln(10)}}}{{\upmu }}$$

5 $${\text{HVL = }}\frac{{\text{Ln(2)}}}{{\upmu }}$$

Using Eq. (6) to calculate the Zeff values^36^.

6 $${\text{Zeff = }}\frac{{\mathop \sum \nolimits_{{\text{i}}} {\text{fiAi(MAC)i}}}}{{\mathop \sum \nolimits_{{\text{i}}} \frac{{{\text{Ai}}}}{{{\text{Zi}}}}{\text{(MAC)i}}}}$$

The weight fraction, atomic weight, and atomic number of each element in a specimen are determined by F_i_, A_i_, and Z_i_ respectively.

### Neutron attenuation

Is described by the neutron removing cross-section (ΣR), which is the probability of neutron reactions within a material, and is given by Eq. (7)^37^:

7 $$\sum R = \sum\limits_{i} {\rho_{i} \left( {\sum R /\rho } \right)_{i} }$$

where (ρi) is the partial density, and (ΣR/ρ) is the mass removal cross-section, which can be calculated for any compound using Eq. (8)^37^:

8 $$\frac{\sum R }{\rho } = 0.206{{A^{{{\raise0.7ex\hbox{${ - 1}$} \!\mathord{\left/ {\vphantom {{ - 1} 3}}\right.\kern-0pt} \!\lower0.7ex\hbox{$3$}}}} } \mathord{\left/ {\vphantom {{A^{{{\raise0.7ex\hbox{${ - 1}$} \!\mathord{\left/ {\vphantom {{ - 1} 3}}\right.\kern-0pt} \!\lower0.7ex\hbox{$3$}}}} } {Z^{ - 0.294} }}} \right. \kern-0pt} {Z^{ - 0.294} }}\;({\text{cm}}^{2} /{\text{g}})$$

where (A) is the atomic weight, and (Z) is the atomic number.

### Monte Carlo Fluka simulation

**Figure 3:**
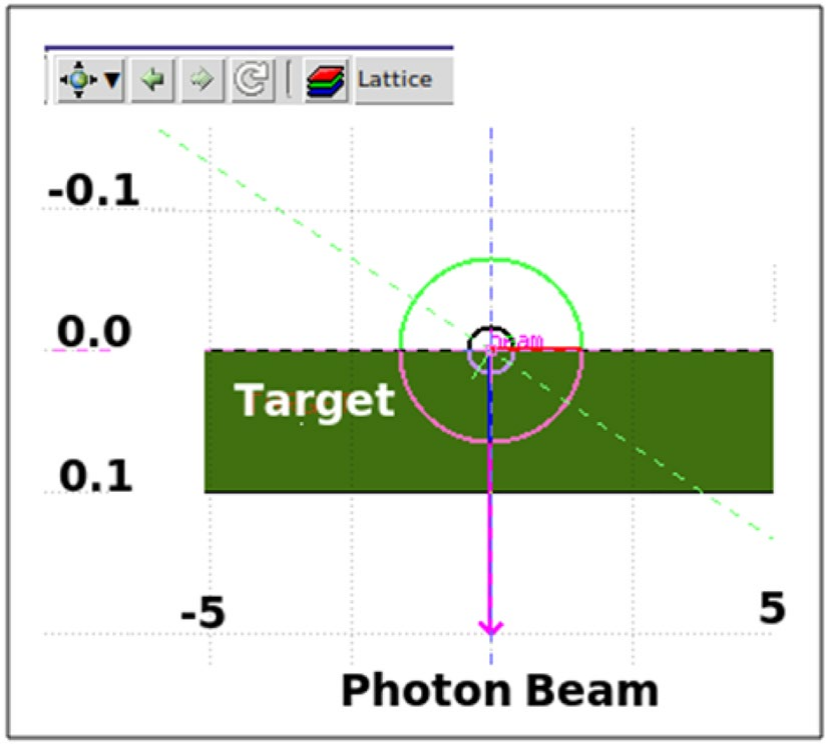
Visual image of the used gamma rays shielding system in the FLUKA code.

**Figure 4:**
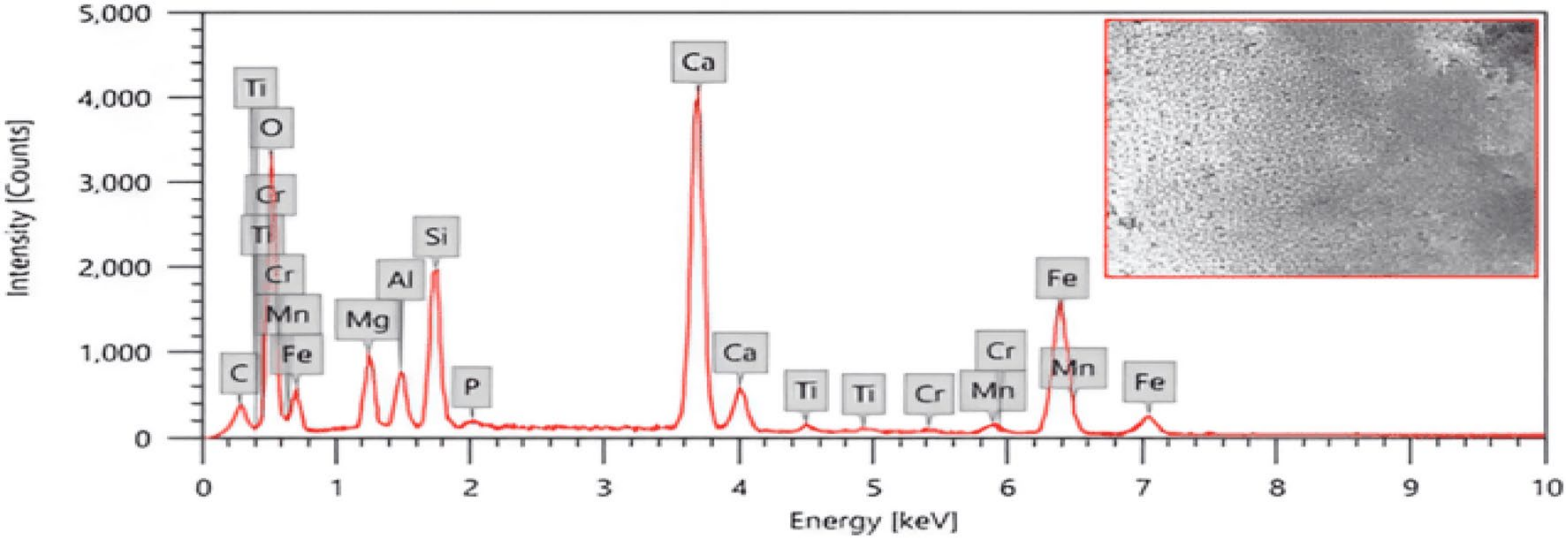
The EDX—analysis and image of present iron slag.

The FLUKA program is a program that simulates the interactions and transportation of particles and photons in matter^37–40^. The shielding characteristics were investigated in this work using the Fluka tool for the investigated samples 0 wt%, 5 wt%, 15 wt% and 25 wt% iron slag samples against γ-rays. In the current simulation, it was assumed that the beam profile was rectangular and that the beam was stationary in the positive z-axis direction. The built-in materials and compound cards have been used to describe sample materials, with elements shown in Fig 4. The model for the sample consisted of a cylinder with a diameter of 10.0 cm and various thicknesses, ranging from 0.1 to 0.5 cm. Simulation processes run for 106 primary photons to obtain a statistical error of <1%. Figure 3 displays the geometry that was simulated with FLUKA, USERDUMP card (method) was used for driven output by calling the provided mgdraw.f routine.

## Results and discussions

### EDX analysis

In this study, the primary material used was IS. Production of nanostructured IS: using a ball mill, the IS was crushed and then ground into a fine powder. To determine the elemental composition quantitatively of the iron slag, an energy dispersive X-ray (EDX) analysis was conducted. The results, including the average mass percentage of each, metal tableted in Table 3. The shape of its particles was confirmed with an SEM “scanning electron microscope Fig. 4^41,42^^.^

**Table 3:** The average mass percentage of each metal in IS powder.

Element	Mass %
C	3.75 ± 0.06
O	39.97 ± 0.27
Mg	3.61 ± 0.07
Al	2.45 ± 0.05
Si	6.55 ± 0.08
P	0.23 ± 0.02
Ca	21.24 ± 0.14
Ti	0.55 ± 0.03
Cr	0.41 ± 0.03
Mn	0.97 ± 0.05
Fe	20.26 ± 0.18
Total	100

**Figure 5:**
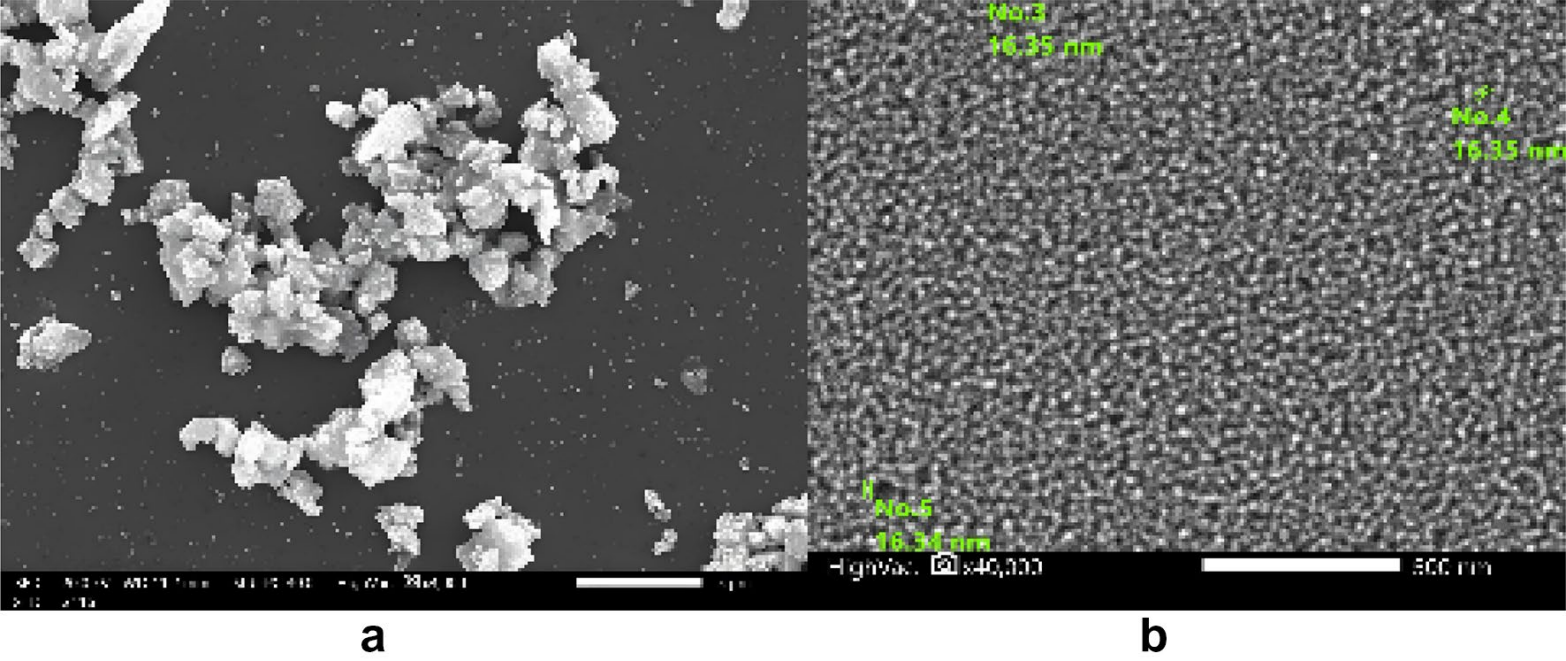
**a.** TEM images of ISMPs. **b.** TEM images of ISNPs.

In order to investigate the structural and analytical characterization for ISMPs and ISNPs, we utilized a transmission electron microscope to conduct the TEM investigation. Figure 5a,b reveals that the average size of microparticles is 3 µm, while nanoparticles measure 16 nm. As we can see in Fig. 5a, it is evident that the majority of the slag particles are larger in size. From Fig. 5b, the large slag particle has been destroyed and crushed by the intense impacts of the balls during milling.

### Effect of iron slag on cell morphology and density

**Figure 6:**
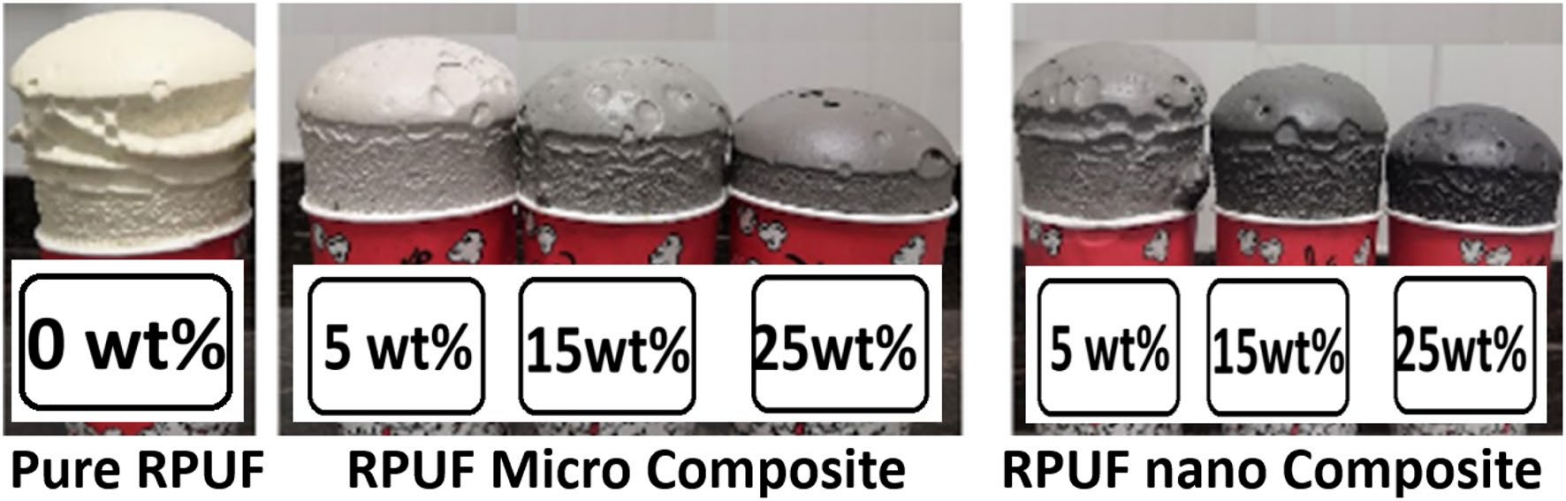
Images of polyurethane foam doped with different percentage of IS filler.

The pure polymer was compared to the polymer mixed with ISMPs and ISNPs displayed in Fig. 6. The polymer doped with ISNPs decreased the volume of the neat sample; however, increasing the quantity of filler to 5 wt%, 15 wt%, and 25 wt% caused the volume has changed. This was due to nanoparticles' larger surface area as compared to microparticles. This benefit of nanoparticles aids in ensuring homogeneous dispersion during preparation via polyurethane foam porosity and eliminates the likelihood of aggregation in the case of microparticle size.

**Figure 7:**
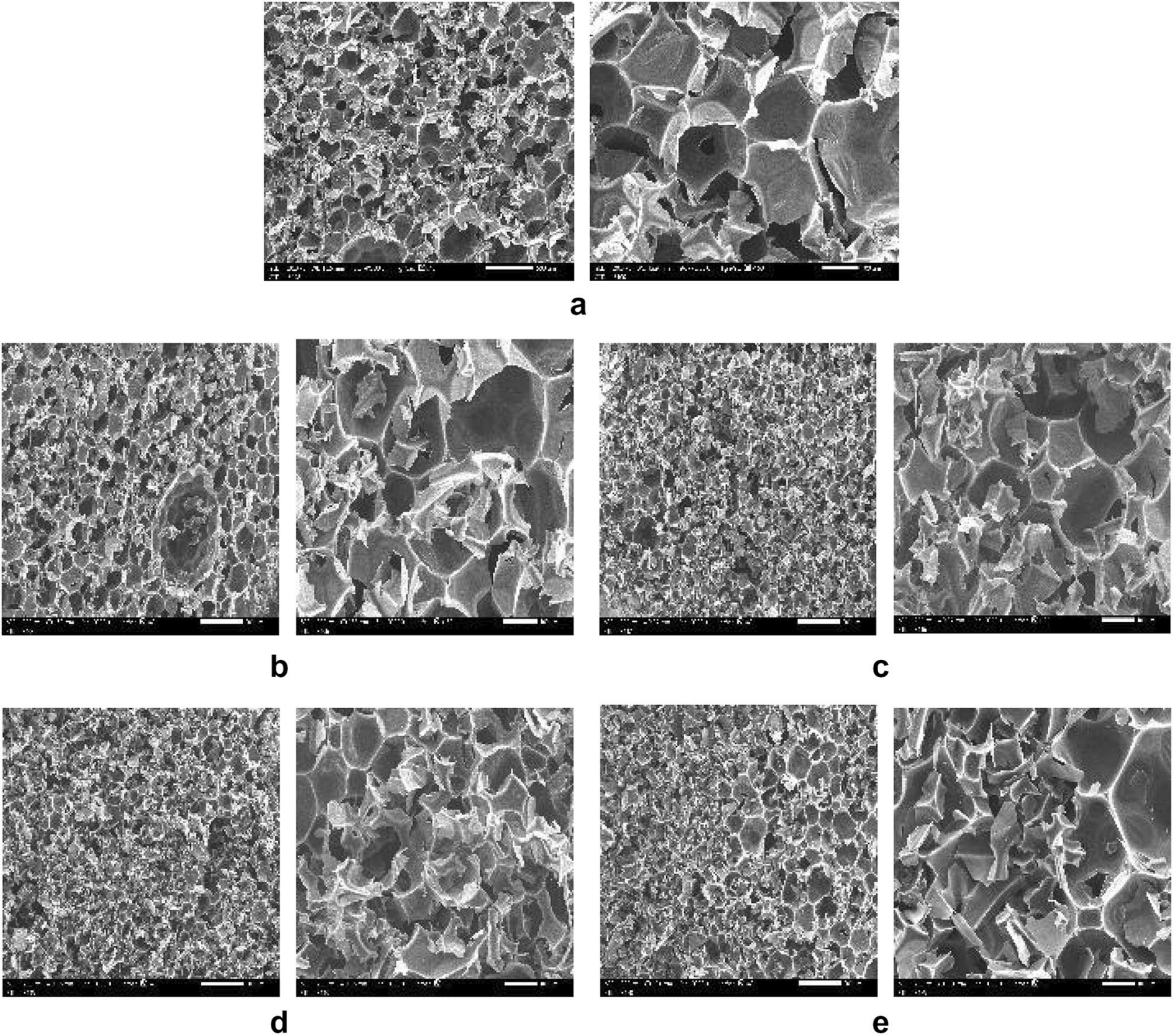
(**a**) SEM of Polyurethane Pure Foam, (**b**) SEM of polyurethane foam 5wt% ISMPs Mixture, (**c**) SEM of polyurethane foam 5wt% ISNPs Mixture, (**d**) SEM of polyurethane foam 25wt% ISMPs Mixture, (**e**) SEM of polyurethane foam 25wt% ISNPs Mixture.

Under a similar reaction technique, two types of composite foams were generated: the first was a polyurethane composite with ISMPs, and the second was a polyurethane composite with ISNPs. The morphological qualities of the prepared nano and micro composite foams were compared to those of neat foam. SEM was used to study the cell morphology of nanocomposite foams, as shown in Fig. 7a–e with two different magnifications: 40 times and 150 times. When we added nanofillers to foams, the cell density increased but the cell size decreased when compared to a pure foam sample. The cell nucleation process has a significant impact on the shape of the final foam and, therefore, plays an essential role in foam generation. Cell nucleation is classified into two forms according to nucleation theory: homogeneous nucleation and heterogeneous nucleation. When no impurities are present in the mixture, homogeneous nucleation occurs. Heterogeneous nucleation occurs in the polymer matrix in the presence of nucleating agents. It happens at the point when the polymer/gas solution meets the nucleates. In fact, nanoparticles and microparticles operate as nucleation agents, increasing cell nucleation by lowering the energy required to create bubbles. Because of their extremely tiny dimensions and high surface density, nanoparticles have far more surface interaction with the polymer matrix and gas molecules than typical nucleating agents. This means that the optimal nanoparticle content can be obtained with a low nominal particle concentration. As a result of the high nucleation that results in a larger number of cells in foams, including nanoparticles, the CO_2_ created from an interaction between water and isocyanate must be used to develop a greater number of cells. Cells that will be culminated into smaller cells, The addition of filler particles in the polymer matrix increases the amount of hydrogen bonding between urethane chains, resulting in poorer polymer flexibility and cell coalescence. Because polymers are less flexible, it is more difficult to produce the gas (carbon dioxide), resulting in smaller cell sizes. An increase in the viscosity of the polymer mixture (when good dispersion had taken place) could result because of limited cell expansion and a decrease in cell size. To compare the effect of nanoparticle levels on foam morphology, two different contents for each ISNPs were selected: 5wt% and 25wt%, and also for ISMPs, 5wt% and 25wt% were selected. The effect of filler particle contents on foam density is tabulated in Table 4. In comparison to neat foam, lower cell size and higher cell density were obtained by the incorporation of filler particles (ISMPs and ISNPs). However, from a low to a high content of ISMPs in the polyurethane mixture, an increase in the mixture density (from 44.13 to 53.24 kg/m^3^). In the case of ISNPs in the polyurethane mixture, This pattern was consistent, in which the mixture density increased from 41.67 to 49.80 kg/m^3^, with an increase in the ISNPs content from 5 to 25wt%. These observations can be related to weak dispersion in high-content nanoparticles. According to SEM micrographs shown in Figs. 7a–e, aggregations of larger size take place in the case of ISMPs in comparison to ISNPs.

**Table 4:** Densities before and after added IS filler.

Sample Name	Density kg/m^3^
Pure	34.60
5 wt % ISMPs	44.10
15 wt % ISMPs	46.40
25 wt % ISMPs	53.20
5 wt % ISNPs	41.70
15 wt % ISNPs	43.20
25 wt % ISNPs	49.80

### Effect of slag on compressive strength

The RPUF was reinforced with different ratios of ISMPs and ISNPs (0–25 wt%). The compression test was conducted using a range of 0–50% of the overall thickness of the sample. The results revealed that by adding IS particles, the compression strength of RPUF was enhanced compared to the neat foam. The load force is applied in a direction parallel to the direction of the foam rise. The compressive strength of foams containing 5 wt% and 25 wt% of either ISMPs or ISNPs fillers increases compared to neat RPUFs., The greatest increase in compressive strength was in the 5 wt% ISMPs sample. For all samples, as we add IS fillers, the compression strength decreases, in which case resistance tends to decline, and for all ISMPs filler samples, it is greater than ISNPs filler at their corresponding concentrations, which is consistent with the density results. As the percentage of filler increases, the compression strength decreases, as shown in Table 5. The decreases of the strength at higher filler concentrations could be attributed to the cell irregularity caused by imperfections in the struts due to the IS filler presence. The compression results of RPUF nano composite samples decreased much more than those of RPUF micro composite samples, which may have been caused by both chemical and physical factors. Due to the cell geometry imperfection, higher density, and thicker struts, rebound resilience also decreased.

**Table 5:** Shows the compressive strength (MPa) with different compressive percentages.

Compressive strength (MPa) with different compression percentage					
Compression percentage	10%	20%	30%	40%	50%
Pure	0.204	0.218	0.236	0.246	0.258
5wt% ISMP	0.289	0.311	0.323	0.333	0.343
15wt% ISMP	0.273	0.298	0.317	0.334	0.348
25wt% ISMP	0.256	0.279	0.296	0.312	0.325
5wt% ISNP	0.253	0.271	0.291	0.306	0.326
15wt% ISNP	0.233	0.254	0.267	0.280	0.290
25wt% ISNP	0.218	0.229	0.253	0.264	0.272

### Thermo-gravimetric analysis (TGA)

**Figure 8:**
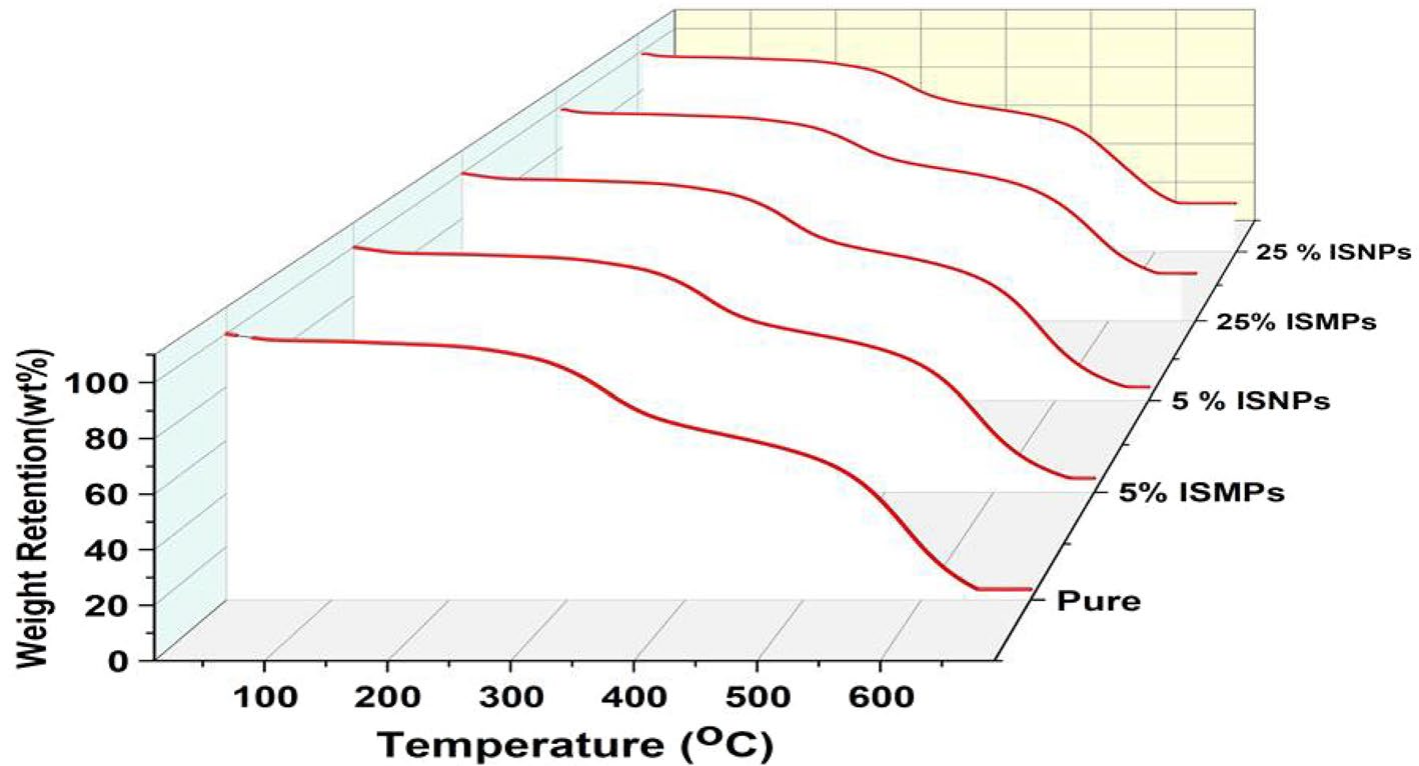
TGA curves for pure RPUF and RPUF composite.

To characterize the thermal stability of the sample, thermogravimetric analysis (TGA) was used. Each sample was underwent to thermogravimetric measurements performed under the following operational conditions; heating rate 10 °C min^−1^, in the temperature range of 10–700 °C. Figure 8 shows the control group (neat RPUF) and RPUR composite samples with different types of fillers (ISMPs and ISNPs). The experiments were conducted under a nitrogen atmosphere. The control group had the least amount of carbon residue. The degradation process had two stages. In the first stage, between 300 and 350 °C, the isocyanate hard segments degraded and the polyurethane molecular chain broke down due to alcohol evolution, provided that enough energy was available. In the second stage, between 450 and 500 °C, the urea linkages and polyol bonds cleaved, which corresponded to the degradation of the polyurethane soft segments, and CO was produced, leading to complex products. The addition of fillers increased the weight loss of polyurethane foam with different filler content ratios. Figure 8 displays the results, while Table 6 exhibits the variations in the decomposition rates. RPUF containing 5 wt% ISMPs and ISNPs showed that they were almost identical. The most carbon residue was observed in RPUF which contained 25 wt% ISMPs and ISNPs. Figure 8 and Table 6 show the changes in the decomposition curves. Td10 is the temperature when the weight loss of the sample reaches 10% for neat RPUF, which was 277 °C when the weight loss was 10%. After IS filler was added to RPUF, and as the concentration of IS increased, Td10 increased considerably to 296°C (25 wt% ISMPs composite) when the weight loss of the hybrid material was 10%. As the concentration of IS increased, Td20 is the temperature at which the weight of the sample loss reached 20% of the neat RPUF, was 236 °C when the weight loss was 20%. Td20 increased considerably to 296°C (25 wt% ISMPs composite).

**Table 6:** shows the initial decomposition temperature (IDT), weight loss at 10%, 20%, and 700 °C for RPUF composite samples.

RPUF	IDT (°C)	Temperature ± 2 °C		Weight loss %at 700 °C
		Weight loss 10%	Weight loss 20%	
Pure	171	277	326	96
5 wt% ISNPs	194	284	333	91
25 wt% ISNPs	310	290	338	76
5 wt % ISMPs	184	284	333	91
25 wt % ISMPs	209	296	347	77

The derivative thermogravimetric curves are displayed in Fig. 8. As the temperature increased and the concentration of IS increased, the weight loss percentage of the composite samples decreased; the weight loss was 96% for the neat RPUF at a temperature of 700 °C. As the concentration of IS increased, the weight loss percentage decreased at the same temperature. The minimum weight loss percentage for the sample of 25 wt% ISMPs was 77%.

After adding IS, it effectively slowed down the degradation rate. So, adding IS could effectively improve the thermal stability of the RPUF composite.

### XRD analysis

**Figure 9:**
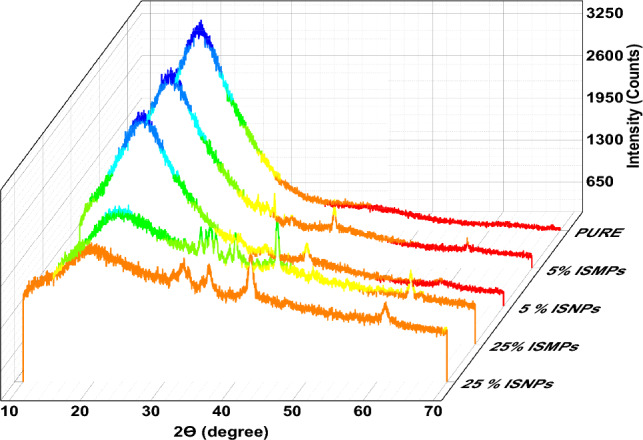
XRD- Curves of Pure RPUF and RPUF composite.

X-ray diffraction (XRD) was utilized to analyze the RPUF composite samples. The XRD in Fig. 9 displays a wide diffraction peak that does not have any discernible sharp peaks, showing that there is a molecular structure that is amorphous. The RPUF materials are compatible with this microstructural trait. Around 20 degrees is where the wide diffraction peak in this material is visible, this matches with observations made with other polyurethane materials. It was demonstrated that, for both ISMPs and ISNPs, the broad peak that denotes the presence of polyurethane foam somewhat decreased when 5wt% IS was added, but the peak vanished when 25wt% IS was introduced. It can be shown, the β-slag substrate is responsible for a number of remarkable peaks at 28.8°, 29.03°–30.00°, 32.40°–36.21°, 39.06°–41.81°, and 61.10°^43^. Compared to 25 wt% ISNPs, the resulting peaks of 25 wt% ISMPs are narrower at half maximum and have sharper peaks. There have been earlier reports of comparable XRD patterns for raw steel slag^44–49^. The XRD profile of polyurethane composite samples with IS exhibited a very complicated composition with several overlapping peaks resulting from various minerals, which can be found in Table 7, including Ca(OH)_2_, Ca_2_Fe_2_O_5_, Ca_3_-Mg(SiO_4_)_2_, Ca2SiO4, etc. With the increase in IS percentage loading, the XRD profile of the foam presented more diffraction peaks compared to raw iron slag. The most abundant mineral phase present in BOF slag is portlandite (Ca(OH)_2_). This mineral is predicted since BOF slag includes 39% lime (CaO), which changes to Ca(OH)_2_ in the presence of moisture. Merwinite (Ca_3_Mg(SiO_4_)_2_) and srebrodol'skite (Ca_2_Fe_2_O_5_) were the two significant phases. The presence of free lime (CaO) proved that Ca(OH)_2_ was transformed into CaO at high temperatures. Water vapor was generated during this reaction to dilute the combustible volatiles during burning.

**Table 7:** Mineral phases discovered in BOF slag.

Mineral type	Formula	BOF Slag
Lime	CaO	Major
Portlandite	Ca(OH)_2_	Major
Larnite	Ca_2_SiO_4_	Major
Merwinite	Ca_3_Mg(SiO_4_)_2_	Major
Srebrodol’skite	Ca_2_Fe_2_O_5_	Major
Dolomite	CaMg(CO_3_)_2_	Major
Calcite manganoan)	(Ca, Mn)CO_3_	Major
Periclase	MgO	Probable
Pentahydrite	CuSO_4_.5H_2_O	Probable
Monticellite	CaMgSiO_4_	Probable
Hematite	Fe_2_O_3_	Probable
Magnesite	MgCO_3_	Probable
Wollastonite	CaSiO_3_	Probable

### FTIR analysis

**Figure 10:**
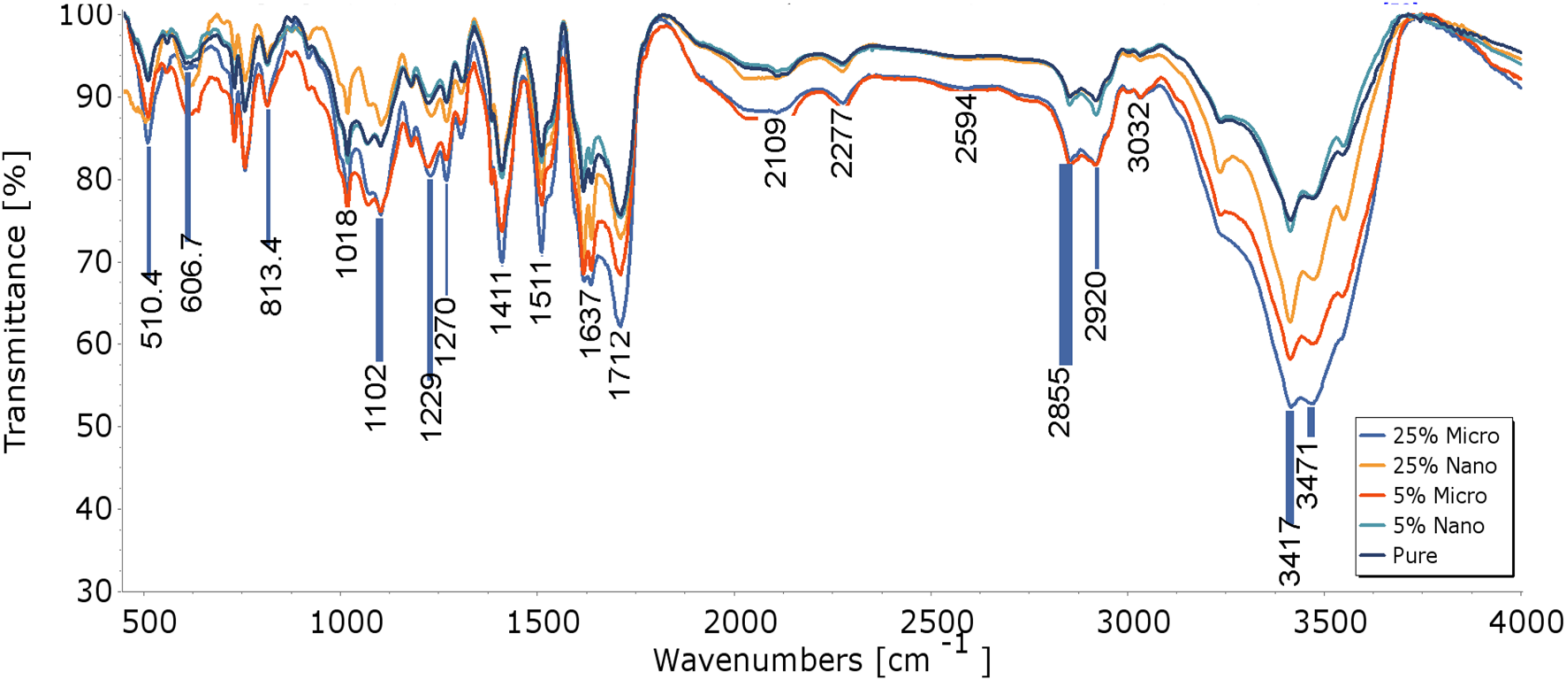
FTIR- Curves of Pure RPUF and RPUF composite.

The PU linkage is produced by the reaction of the hydroxyl functionalities of the polyol (O–H) with an isocyanate group (N=C=O). Any hydrogen atoms attached to atoms are more electronegative than carbon active for the reaction with isocyanates. Considering this fact, the incorporation of nanoparticles leads to a reaction between nano slag surface hydroxyl groups and NCO groups of isocyanates. The FTIR spectroscopy result of rigid PU foam in air at room temperature (RT) is shown in Fig. 10. (Spectral range: 4500–500 cm^−1^). The stretching vibration characteristic peak of –OH at 3417 cm^−1^, peaks appear at 2855 and 2920 cm^−1^, which are the symmetric and antisymmetric absorption peaks of –CH_2_, peaks at 1712 and 1637 cm^−1^ indicate the presence of C=O and C=C functional groups, the absorption peaks appear at 1511, 1299, 1102, and 1102 cm^−1^, which are attributed to C–N, N–H, and Si–O–Si absorption, respectively^50^,Bond stretch results in a broad and strong peak at 1018 cm^−1^.where this results in good agreement with Assignment^51^ as shown in the following Table 8. The iron slag FTIR spectra display different absorption bands at 3432, 1633, 1434, 1112, 986,873 and 708 cm^−1^. The band at 3432 cm^−1^ belongs to the stretching vibration of the hydroxyl group caused by the weakly absorbed water molecules on the slag surface, while the band at 1633 cm^−1^ is representative of portlandite^52^. The peculiar absorption bands at 1434, 873 and 708 cm^−1^ are assigned to the asymmetric stretching mode and bending mode of the carbonate group, respectively^39^. The bands at 1112 and 986 cm^−1^ are caused by Si–O stretching vibrations^53^.

**Table 8:** Shown the Assignment of FTIR spectrum of RPUF.

Wave number (cm^−1^)	Assignment	Wave number (cm^−1^)	Assignment
3471	Phenols Ar O–H H-bonded	1511	N–O nitro comp + N–H Amide + (C-N)
3417	R3C-OH O–H stretch	1411	(C = C) isocyanurate rate ring
3032	C-H aromatic ring	1270	C-H , (-CH_2_X)
2920	(C-H) in CH_2_	1229	N–O nitro comp + N–H Amide + (C-N)
2594	(O =) PO-H phosphonic acid	1102	Si–O-Si
2277	(N = C = O) in isocyanate	1018	(C–O–C) in Urethane O = C–O–C
2109	(N=C=N) in isocyanate	813.4	P(C-H) in aromatic
1712	(CO) in ester, Urethane H bonded,	757.2	RCO-OH carbolic acids
	Retronecine carboxylic acid	606.7	Sharp spike due to CO_2_
1637	(C=C) aromatic	510.4	Ring bending band

### Experimental results of attenuations coefficients

Iron slag typically contains some elements listed in Table 3, some of which have a high atomic number. These elements have a strong interaction with gamma radiation, resulting in effective attenuation^54–56^.

### LAC and MAC results

**Figure 11:**
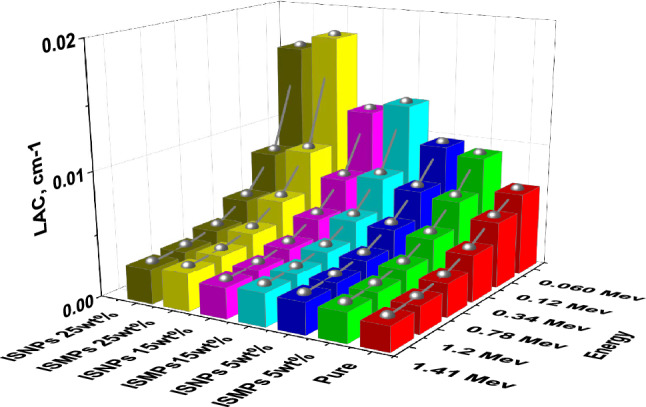
The LAC of RPUF reinforced with ISMPs and ISNPs in in different broad energy.

Based on calculated the LAC for all the composite samples based on the experimental results. Table 9 and Fig. 11 show the results. We also calculated the MAC using the sample densities and showed the results in Table 10. To verify the accuracy of these experimental results, we used XCOM and FLUKA tools to calculate the MAC and compare it with the experimental MAC values. Table 10 shows the experimental MAC values for all the composite samples RPUF ISMPs and RPUF ISNPs with different concentration ratios of 5 wt%, 15 wt%, and 25 wt%. The data showed convergence at medium and high energies due to the density effect, the data revealed convergence at medium and high energies. At energy 1.173 MeV, for example, the MAC values for the neat sample are less than those of the micro- and nano-composites samples. The experimental results were verified by using the theoretical MAC given by the computer algorithms WinXCOM^54^ and FLUKA. The XCOM and FLUKA data were compared to the neat RPUF and micro composites, and the relative deviation for LAC did not exceed 6.25% in all discussed energies, results of LAC either experimentally or theoretically are presents in Table 9.

**Table 9:** The LAC by experimental results and XCOM of RPUF composites.

			LAC, cm-1											
Energy (MeV)	Pure RPUF		RPUF + ISMPS									RPUF + ISNPS		
			5wt%			15wt%			25wt%			5wt%	15wt%	25wt%
	Exp	XCOM	Exp	XCOM	Dev %	Exp	XCOM	Dev%	Exp	XCOM	Dev%	Exp	Exp	Exp
0.059	0.0066	0.0065	0.0098	0.0097	0.68	0.0125	0.013	− 5.42	0.0173	0.0169	6.61	0.0097	0.0125	0.0178
0.081	0.006	0.0059	0.0082	0.0081	1.13	0.0095	0.0097	− 2.54	0.012	0.0115	3.64	0.0081	0.0095	0.0121
0.122	0.0053	0.0052	0.007	0.0068	6.61	0.0075	0.0075	0.32	0.0089	0.009	− 1.41	0.0069	0.0075	0.0089
0.245	0.0042	0.0042	0.0055	0.0053	3.64	0.0057	0.0055	4.18	0.0065	0.0069	− 5.42	0.0054	0.0057	0.0065
0.344	0.0037	0.0037	0.0049	0.0049	− 1.41	0.005	0.0049	2.36	0.0057	0.0058	− 2.54	0.0047	0.0050	0.0057
0.662	0.0028	0.0029	0.0037	0.0039	− 5.42	0.0038	0.0038	0.68	0.0043	0.0044	− 5.42	0.0036	0.0038	0.0043
0.779	0.0026	0.0027	0.0034	0.0035	− 2.54	0.0035	0.0035	1.13	0.004	0.0041	− 2.54	0.0034	0.0035	0.0040
0.964	0.0024	0.0023	0.0031	0.0031	0.32	0.0032	0.003	6.61	0.0036	0.0036	0.32	0.0030	0.0032	0.0036
1.173	0.0022	0.0021	0.0028	0.0027	5.18	0.0029	0.0028	3.64	0.0033	0.0032	5.18	0.0028	0.0029	0.0033
1.333	0.002	0.002	0.0026	0.0026	2.36	0.0027	0.0027	− 1.41	0.0031	0.003	2.36	0.0026	0.0027	0.0031
1.408	0.002	0.002	0.0026	0.0025	2.32	0.0026	0.0027	− 4.42	0.003	0.0029	1.13	0.0025	0.0026	0.0030

**Table 10:** The MAC of RPUF reinforced with ISMPs and ISNPs at different broad energies.

Energy (MeV)	MAC, (cm2.g^−1^)						
	Pure RPUF	Pure RPUF + ISMPS			Pure RPUF + ISNPS		
		5%	15%	25%	5%	15%	25%
0.059	0.1907	0.2221	0.2697	0.3252	0.2323	0.2896	0.3575
0.081	0.1723	0.1867	0.2044	0.2245	0.1942	0.2195	0.2437
0.122	0.1530	0.1592	0.1620	0.1667	0.1651	0.1739	0.1792
0.245	0.1224	0.1251	0.1229	0.1224	0.1295	0.1320	0.1307
0.344	0.1079	0.1101	0.1079	0.1067	0.1139	0.1158	0.1140
0.662	0.0822	0.0838	0.0818	0.0808	0.0868	0.0879	0.0862
0.779	0.0764	0.0778	0.0759	0.0750	0.0805	0.0816	0.0800
0.964	0.0690	0.0703	0.0686	0.0677	0.0728	0.0737	0.0723
1.173	0.0626	0.0638	0.0623	0.0615	0.0661	0.0669	0.0656
1.333	0.0587	0.0598	0.0583	0.0576	0.0619	0.0626	0.0614
1.408	0.0570	0.0581	0.0567	0.0560	0.0601	0.0609	0.0597

The mass attenuation coefficient was determined by uses Eq. (2) and compared with those values at the same energies obtained from XCOM software. From the theoretical MAC obtained from XCOM software and by knowing the density, LAC values have been calculated for the present composites, as shown in Fig. 11. From the figure, the inclusion of IS in the matrix improved the composites' overall attenuation abilities. LAC of 25 wt% IS > LAC of 15 wt% IS > LAC of 5 wt% IS > LAC of pure RPUF. If we compared the effects of ISMPs and ISNPs in the matrix, we observed that the effect of ISMPs slightly enhanced the shielding ability more than of ISNPs when adding the 5wt% percentage of IS. With increasing the percentage of IS, the effect of ISNPs enhanced the shielding ability more than of ISMPs. This is related to the density effect. and particle distribution together. The greater the amount of IS, the greater the distribution and homogeneity within the matrix, and thus the greater the attenuation coefficient and the better shielding ability, as shown in Table 10. The photoelectric effect is the dominating interaction of photons at low energy, whereas Compton scattering is dominant for photons with energy more than 200 keV up to 2 MeV. Compton scattering will compete with the pair formation interaction at higher energy levels. At higher levels, the pair production interaction will be dominating.

**Figure 12:**
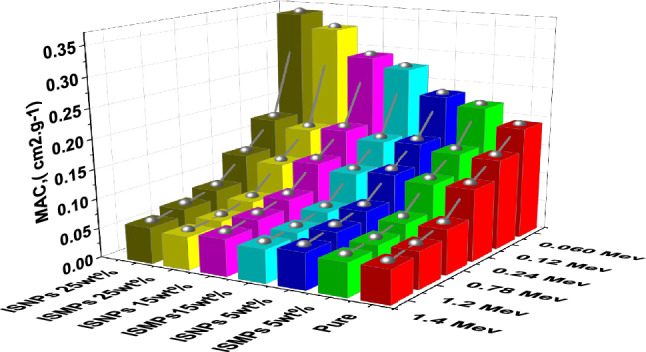
The MAC of RPUF reinforced with ISMPs and ISNPs in in different broad energy.

Furthermore, as demonstrated in Table 10, Fig. 12, the MAC values for nanocomposites are larger than those for micro composites for all energies and IS percentages (5 wt%, 15 wt%, and 25 wt%). These results are attributed to the larger dispersion of IS nanoparticles in the RPUF matrix than IS microparticles, since a better distribution improves the composite's attenuation ability.

### Relative change of LAC for RPUF reinforced with ISMPs and ISNPs

The relative change for the LAC of RPUF which reinforced with ISM and ISPs at different broad energies, and the relative change can be calculated using Eqs. (9–10) as shown in Table 11.

9 $$\varvec{Relative\, change}\left( {{\varvec{micro}}} \right) = \left( {\left( {{\varvec{LAC}}_{{{\varvec{ISMPs}}}} - {\varvec{LAC}}_{{{\varvec{Pure}}}} } \right)/{\varvec{LAC}}_{{{\varvec{Pure}}}} } \right) \times 100,\user2{\% }$$

10 $$\user2{Relative\, change }\left( {{\varvec{nano}}} \right) = \left( {\left( {{\varvec{LAC}}_{{{\varvec{ISNPs}}}} - {\varvec{LAC}}_{{{\varvec{Pure}}}} } \right)/{\varvec{LAC}}_{{{\varvec{Pure}}}} } \right) \times 100,\%$$

**Table 11:** The relative change of LAC of RPUF reinforced with ISMPs and ISNPs at different broad energies.

Energy Mev	The relative change LAC, cm^-1^					
	Pure + 5wt% ISMPS (%)	Pure + 5wt% ISNPS (%)	Pure + 15wt% ISMPS (%)	Pure + 15wt% ISNPS (%)	Pure + 25wt% ISMPS (%)	Pure + 25wt% ISNPS (%)
0.059	48.75	46.92	89.94	89.92	162.74	170.19
0.081	38.40	35.94	59.32	59.32	100.75	103.86
0.122	32.90	30.15	42.20	42.14	67.87	68.81
0.245	30.54	27.61	34.85	34.87	54.07	53.90
0.344	30.33	27.32	34.30	34.21	52.36	52.28
0.662	30.21	27.36	33.65	33.73	51.45	51.14
0.779	30.06	27.08	33.42	33.57	51.25	50.92
0.964	30.13	27.25	33.52	33.58	51.17	51.02
1.173	30.17	27.35	33.66	33.65	51.36	51.04
1.333	30.12	27.19	33.38	33.37	51.18	50.76
1.408	30.19	27.17	33.59	33.61	51.37	50.96

### HVL results for RPUF reinforced with ISMPs and ISNPs

**Figure 13:**
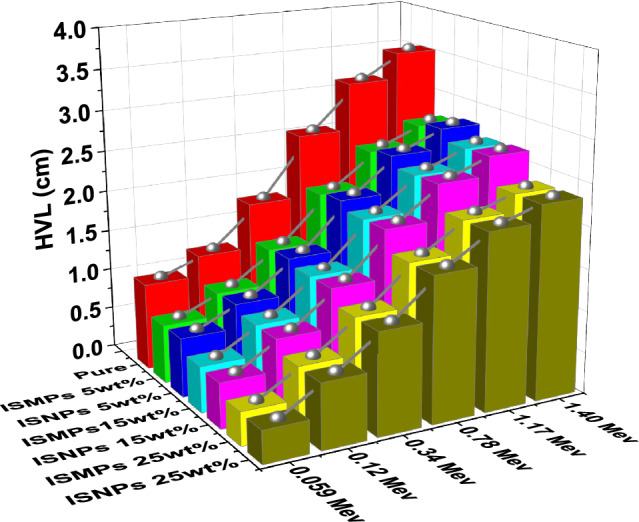
HVL of RPUF reinforced with ISMPs and ISNPs in in different broad energy.

HVL was calculated to estimate the required thickness to attenuate half of incident gamma photons. HVL of the pure RPUF and RPUF IS composites, with a different percentage of IS filler (5wt %, 15 wt%, and 25 wt%) was calculated and shown in Fig. 13. The results showed that the HVL values increased as photon energy increased. The HVL values decrease when the IS percentages in the matrix RPUF composites increase; HVL value at 25wt% ISMPs Composite is the lowest for all discussed energies. In other words, the HVL values of the composites reinforced with ISNPs are lower than those of the ISMPs in all energy ranges except for the 5 wt% IS percentage (Table 12).

**Table 12:** Monomer units, abbreviations and densities of compared polymers^57^.

Polymer	Monomer	Density (g/cm^3^)
Bakelite (PF)	C_9_H_9_O	1.45
Polypropylene (PP)	C_3_H_6_	0.90
Polystyrene (PS)	C_8_H_8_	1.06
Polyethylene (PE)	C_2_H_4_	0.94
Natural rubber (NR)	C_5_H_8_	0.92
Polyvinyl chloride (PVC)	C_2_H_3_Cl	1.30
Rigid Polyurethane foam (RPUF)	C_27_H_36_N_2_O_10_	0.40 (This work)

### MFP results for RPUF reinforced with ISMPs and ISNPs

**Figure 14:**
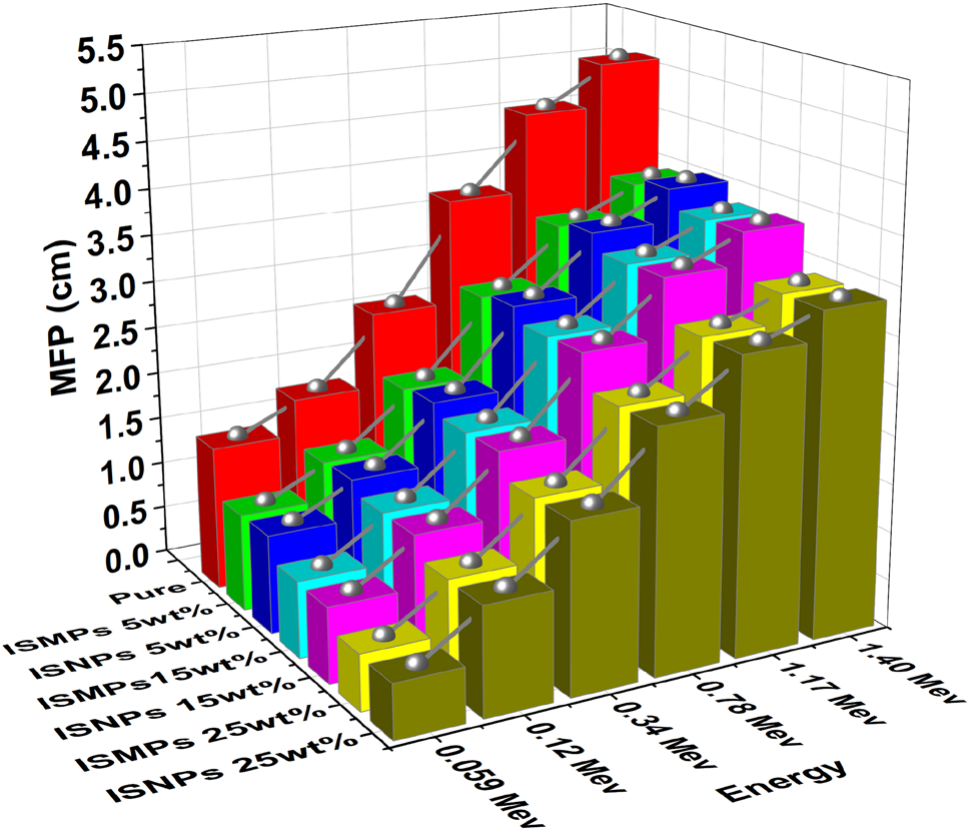
The MFP of RPUF reinforced with ISMPs and ISNPs in in different broad energy.

The average distance traveled by photon between two successive interactions is measured by MEP. The MFP of pure RPUF and RPUF composites with different percentages of IS filler (5 wt%, 15 wt%, and 25 wt%). The results in Fig. 14 shown that as photon energy increased, correspondingly increased the MFP values. MFP values decrease as IS percentages increase in matrix RPUF composites; for example, MFP at 25 wt% ISMPs/RPUF polymer is the lowest value among the related micro-composites at all discussed energies. In other words, except for the 5 wt% IS percentage, the MFP of the composites composite by ISNPs is lower than that of the ISMPs composites in all energy ranges.

### TVL results for RPUF reinforced with ISMPs and ISNPs

**Figure 15:**
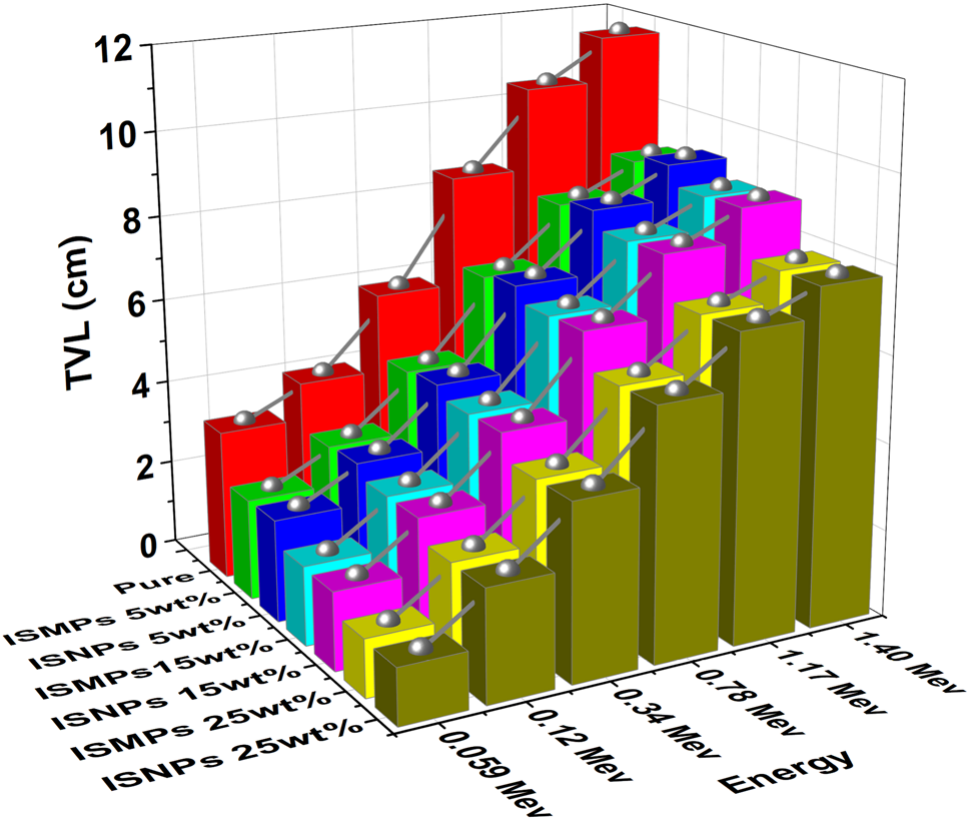
The TVL of RPUF reinforced with ISMPs and ISNPs in in different broad energy.

TVL of the pure RPUF and RPUF composite (5 wt%, 15 wt%, and 25 wt%) was calculated and shown in Fig. 15.

According to the results and as shown in Fig. 15, the TVL values increase with an increase in photon energy. TVL values decrease as IS percentages increase in matrix RPUF composites; for example, TVL at 25wt% ISMPs/RPUF polymer is the lowest value among the related micro-composites at all discussed energies. In other words, except for the 5wt% IS percentage, the TVL of the composites reinforced with ISNPs is lower than that of the ISMPs composites in all energy ranges.

### Zeff results for RPUF reinforced with ISMPs

**Figure 16:**
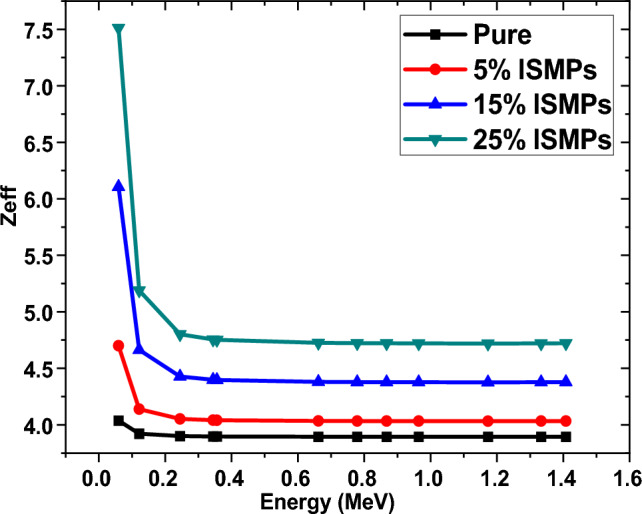
The effective atomic number of RPUF reinforced with ISMPs and ISNPs in in different broad energy.

In order to investigate the shielding effectiveness of RPUF to serve in gamma shielding applications, we also calculated Zeff. Figure 16 illustrates the Zeff values for prepared RPUF composites in relation to photon energy. Increasing photon energy causes decrease in the values of Zeff.

Zeff values increase as IS percentages increase in matrix RPUF composites, Zeff had a higher value at the samples in which 25 wt% IS, and also, as we increase the energy level, the value of Zeff will decrease; for example, energy 0.059 MeV has the highest value of Zeff (7.5) for a 25 wt% composite sample, while at energy 1.4081 MeV, the lowest value for Zeff (2.90) for the neat RPUF.

### Fluka results for RPUF reinforced with ISMPs

**Figure 17:**
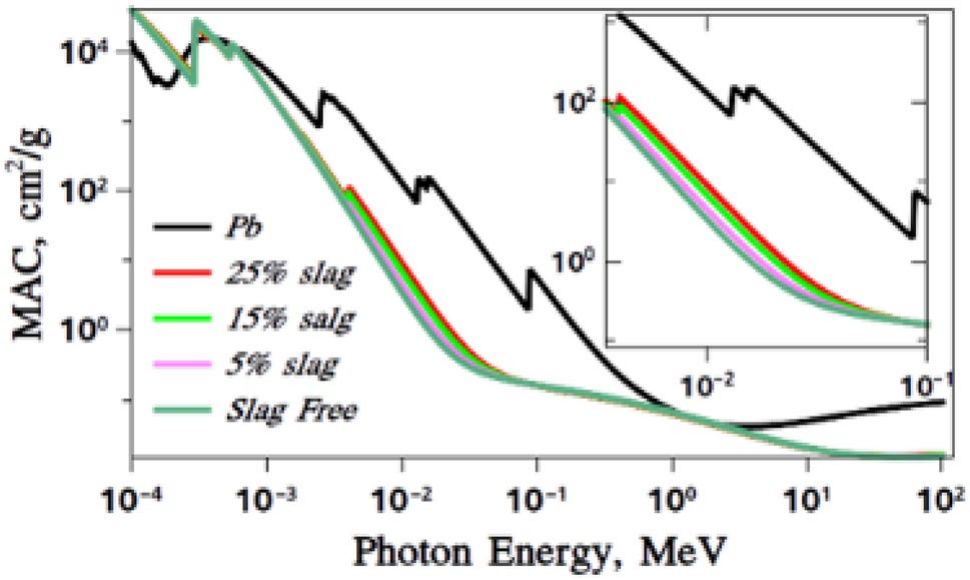
MAC calculated using FLUKA code.

The data from XCOM were validated by comparing them with the data calculated from FLUKA Monte Carlo simulation code. The MAC values for the selected samples were obtained for a wide energy range from 0.0001 to 100 MeV, as shown in Fig. 17.

### MAC obtained results from FLUKA

**Figure 18:**
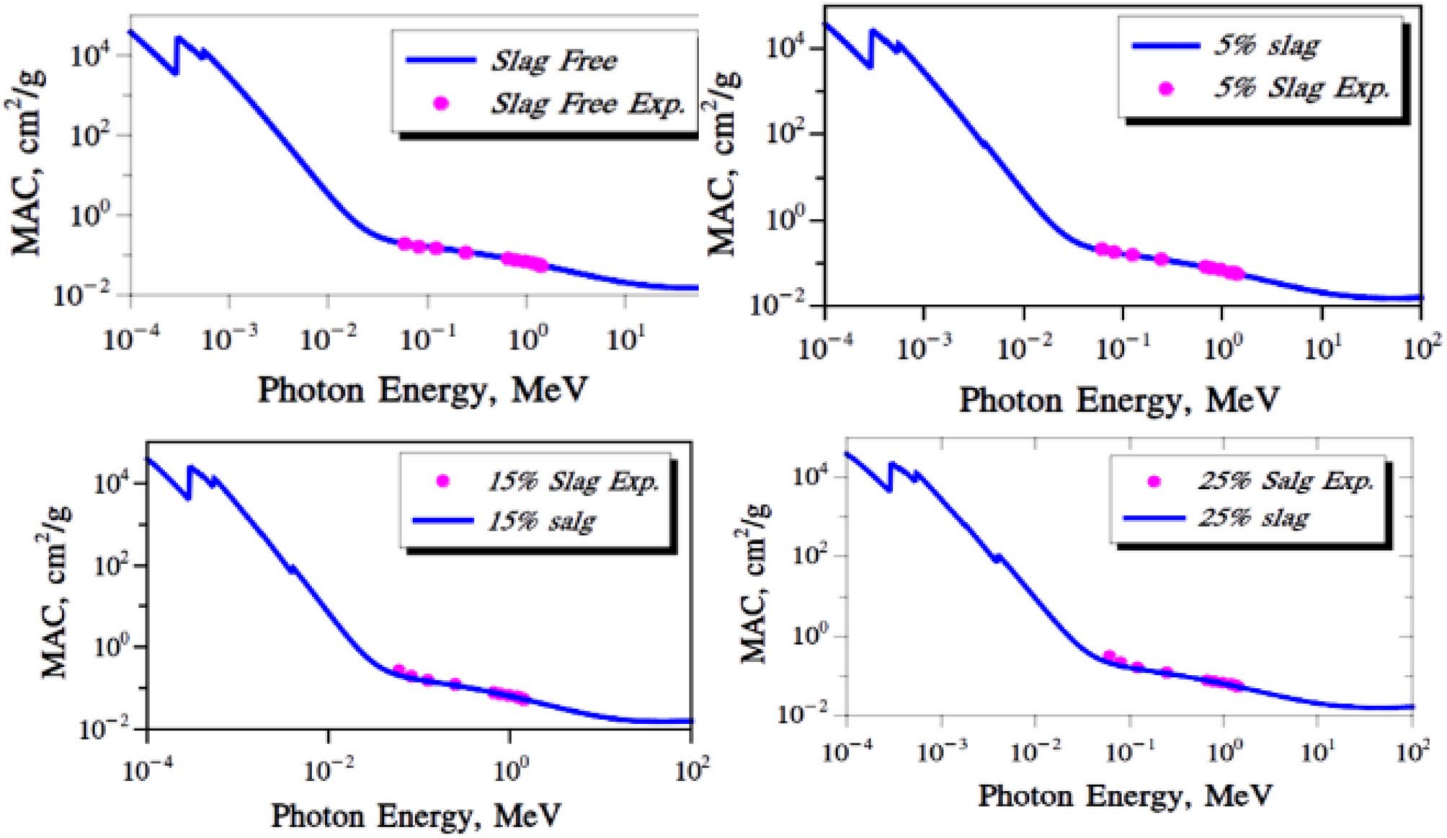
Confirmation of the experimental results using the FLUKA calculations.

The MAC values of all samples moderate sharply with the rise of incident photon energy. In Fig. 18, The zoom-nested Figure shows a non-overlap region from 0.005 up to 0.1 MeV. The curves in these regions are more resolved and clearer. It shows that the 25wt% slag sample has a higher value than the others.

Figure 17 shows that composite slag blocks gamma rays just as well as lead. This blocking works for specific gamma rays, between 0.59 and 3 MeV. Some radioactive materials, like Cs-137 and Co-60, emit gamma rays within this range. Since these materials emit gamma rays at energies 0.59, 1.17, and 1.33 MeV, and a sum peak at 2.5 MeV, composite slag becomes a good alternative for shielding in applications that use these materials. This includes gamma irradiator facilities, blood irradiation, radiobiological research, sterilization, and food irradiation.

### MAC comparing results from XCOM and FLUKA

Comparing the practical results with the calculations of the FLUKA program showed that there is a good match between the two results. This can be shown clearly in Fig. 18.

### Fast neutron removal cross section

**Figure 19:**
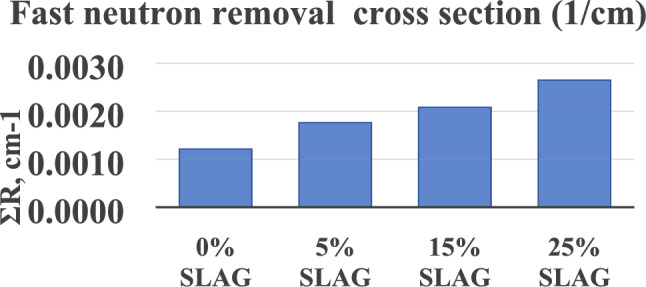
Fast Neutron Removal Cross-Section calculated for (0-15-25 wt%) samples.

In order to test the synthetic material as a shield for fast neutrons, the removal cross-section (∑R) was calculated and given in Fig. 19, the neutron removal cross section are ranging from 0.0012up to 0.0026 cm^−1^. The difference in values is due to those materials containing low-atomic-number elements, which are perfect neutron absorbers. The quantity (ΣR) was calculated by the partial density method^56^.

**Figure 20:**
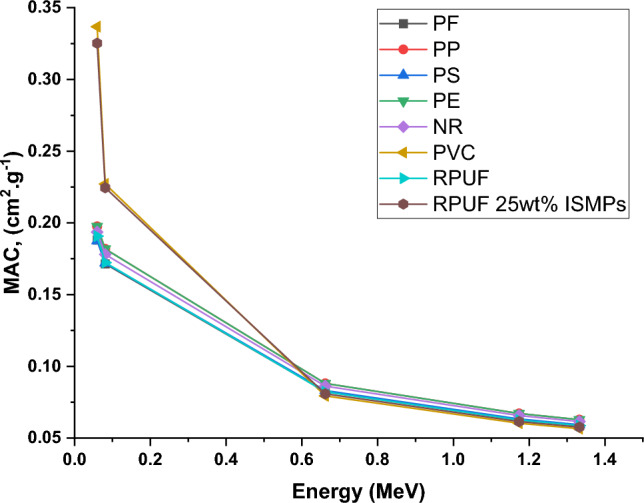
Compression of mass attenuation coefficient values for different similar polymers in references at similar photon energies.

Figure 20 details the mass attenuation coefficients (μm) for various polymers across gamma photon energies, confirmed with XCOM database^58^ ).

## Conclusion

The RPUF composite system in the chemical composition of pure polyurethane foam reinforced with ISMPs and ISNPs powder with a range of 0–25wt% mass by weight has been successfully investigated. The results confirmed that the presence of IS particles enhanced compressive strength compared to the neat foam, and while increasing the IS percentage, compressive strength decreased due to the irregularity of the cells attributed to the presence of IS particles.

The TGA curves of RPUF composite samples indicate the addition of filler consisting of ISMPs and ISNPs significantly affects RPUF degradation and increases the thermal stability of the material, the XRD profile of the foam presented more diffraction peaks compared to raw iron slag. FTIR analysis results show a good agreement with RPUF Assignment.

Gamma-ray shielding properties have been investigated, the Calculated MAC values based on the experimental results of the LAC were in confirmed with those calculated by using WinXCOM program. The results indicated that the values of MAC and Zeff increase and the values HVL, MFP and TVL decrease with increasing IS contents in RPUF sample, then using Fluka simulation code to validate the results in the energy range of photon energies of 0.0001–100 MeV.

The paper also involves studying the interaction of higher-energy radiation (above 20 MeV) with the sample for other high-energy nuclear shielding purposes. This hint helps clarify the suitability of the introduced material for enhanced Gamma Irradiation Protection.

The use of iron slag as a waste to improve the properties of polyurethane, either thermal stability or mechanical properties, as well as a shielding material, is a promising work for research. More attention has to be considered to how to increase the amount of filler in the composite.

Which encourages us to use polyurethane iron-slag composite foam in sandwich panel manufacturing as walls to provide protection from radiation and also heat insulation, due to the ease handling and processes of such materials to give the same effects of ordinary shielding materials e.g. (lead, concrete, … ).

## Data Availability

All data generated or analyzed during this study are included in this published article.
